# The Seven Ages of Man

**Published:** 1888-11-03

**Authors:** 


### THE DOCTOR'S PULPIT.

### THE SEVEN AGES OF MAN.

### V.—THE JUSTICE.

"Then the justice,
In fair round belly with good capon lined,
With eyes severe, and beard of formal cut;
Full of wise saws and modern instances."

SOME people think Shakespeare caricatured the justices of his time because of his own youthful experience with Sir Thomas Lucy. Perhaps he did. There are abundant proofs that the poet was human, very human indeed; and we can very well imagine the more than royal contempt an intellect like Shakespeare's would feel for a mere empty pomposity, grinding out sentences and "cases" got by heart, from so exalted a position as the seat of judgment. Granted, however, a spice of caricature, there is no reason to doubt, but ample evidence to show, that many of the "justices" at the period of the redoubtable Tudors were such as has been described.

The natural qualities that mark a man out for the town council, the alderman's chair, and the justice's bench are very similar in all ages. A certain amount of small personal ambition, a corresponding degree of worldly prosperity, a more than usual share of self-confidence, and the power of learning by heart and uttering like an oracle a few of the commonplace maxims of worldly experience and legal lore—these have furnished a fine equipment for magisterial dignity in all ages and countries from the earliest time until now.

Let it not be imagined that justice and its administration are here held up to cynical contempt. On the contrary, from a patriotic and statesmanlike standpoint, justice is probably the noblest of all achievements to be attained to in any community. That every man, woman, and child in the land shall feel certain in his own mind that the taking of his cause before the highest judge or the humblest magistrate will certainly result in obtaining for it fair play, candour, and full justice—that is an ideal of absolute perfection. Not only is it a "counsel of perfection," but it is an end to be steadfastly and strenuously aimed at by every man who has intelligence enough and a disposition honest enough to comprehend and rightly value the position here affirmed. The man who does not see the full significance of a principle of this kind has a fool's intellect; he who sees but does not give a practical assent to it has a scoundrel's heart.

Shakespeare's "justice," compared with this high, but only right standard, was a sorry fellow. His most characteristic feature was his "stomach," as we should say now. Shakespeare more correctly spoke of it as his "belly." It was indeed his "fair round belly" he meant to describe, and not merely that small portion of it which contained his stomach. His "belly" first, and then what it contained, a good fat "capon."

There can be no adequate reason why a justice should not eat, and eat capon, too, if he likes it. Probably, indeed, he is more likely to do his duty if he eats well than if he be a "lean and hungry" man. A "hungry man," it is said, is an "angry man," and an angry man is by no means fit for the seat of judgment. Anger is the parent of two bad faults, haste and severity. By all means, therefore, let the justice eat. But if he eat too much and too well that may give rise to another source of mischief. Dyspepsia often comes of much good eating, and dyspepsia, it is well known, blinds the eyes of many of its victims, and impairs sweetness of temper in them all. On this account, though the justice is permitted to eat, let him eat "wisely," and not "too well." These words of warning are not superfluous, even at the present time. There are not a few justices both in town and country, both clerical and lay, who still swear too steadfastly by the capon, adding to it not "sack," but something less conducive to sound digestion and the just performance of their functions. The wine and the whiskey-and-water of modern luncheon and dinner tables are often much less wholesome than the "sack" of an earlier period; and the digestion of the nineteenth century is probably much less vigorous than that of the fifteenth and sixteenth. Modern justices as well as Shakespeare's will not be the worse for a little sober eating and drinking as well as sober reflection.

From the stomach to the head; from the prime feature of the justice to the less important and less distinctly characteristic: such is the poet's order of precedence. "Eyes severe, and beard of formal cut." As becomes an actor and a justice, he must take care at least to "look his part." But why must the eyes be necessarily severe? It is not the only function of the justice to condemn. It is not, indeed, his principal function. His chief business is to investigate; to investigate first, thoroughly and carefully, in order that he may ascertain all the facts; to investigate and then to weigh. That is surely a truer portraiture of Justice which represents her as holding a pair of scales in her hand than that which pictures her as blind. The justice whose eyes are "severe," and only severe, carries injustice in his face. The battery of stern words which he so frequently turns upon others should be turned upon himself. Quite recently—within the last week or two—the daily papers have reported numerous instances of mere children sent to herd with criminals in a well-known gaol in the North Riding of Yorkshire, for offences of the most trivial character—a few turnips taken out of a field; a hedge broken down whilst following a paper chase. This is the very stupidity and parody of justice. This is to turn gaols not into houses of correction, but into manufactories of criminals. Justices who do these things must be even more incapable and wicked than Shakespeare's fools. Their "eyes severe and beard of formal cut" should be made familiar with the inside of a prisoner's cell in order that experience may teach them that plain sense and elementary fairness which public schools, universities, and "good society" have alike failed to impart.

"Full of wise saws and modern instances." How dearly a fool loves to play the oracle! It is the nature of an ass to bray; but put him into the town council or on the alderman's or justice's bench, and he will fill both town and country with his "hee-haws." Some readers may think we are unnecessarily severe on the administrators of justice; but let them seriously consider the issues at stake. Civilisation itself, and what is still more important, public and private morality, are in danger of being ruined and destroyed by the foolish or unjust making and administering of laws. What is more shocking than to see a drunkard or an adulterer in the pulpit? It is just as monstrous to place a fool or an unjust man on the magistrates' bench. "Wise saws" are very well in their place, and "modern instances" help to a right decision. But in addition to mere learning and knowledge of cases, there should be in the humblest, as well as in the highest, magistrate and judge, a fair, candid, and impartial mind; a mind naturally capable of investigating and weighing evidence; and not only so, but a mind trained in the methods of the law courts. If the State pays its clergy and its doctors, still more should it pay its magistrates. If we cannot afford to leave street scavenging to untrained volunteers, how much less can we afford to leave the administration of justice between man and man. Of all the advantages which civilisation, science and religion ought to secure for modern peoples, nothing whatever can surpass in value and exceed in importance the making of wise laws, and the administering of them with reason and justice.

